# Neural Correlates of Dual‐Functional Local Dynamic Stability in Older Adults

**DOI:** 10.1002/alz.088668

**Published:** 2025-01-09

**Authors:** Sa Zhou, Feng Vankee Lin

**Affiliations:** ^1^ Stanford University, stanford, CA USA; ^2^ Stanford University, Stanford, CA USA

## Abstract

**Background:**

To maintain local dynamic stability (LDS), both cognitively and physically, without being interfered with by internal or external disturbance is important for functional independence but declines as ages. However, some older adults are capable of high dual‐functional LDS. Understanding the brain differences between these older adults and their counterparts may help discover protective mechanisms for functional independence in old age. The purpose of the study was to compare the brain topology between older adults with heterogenous dual‐functional LDS.

**Method:**

Cognitively and physically healthy older individuals (n = 52) were recruited and categorized into four groups (low cognitive/low physical, low cognitive/high physical, high cognitive/low physical, and high cognitive/high physical LDS groups) based on the intra‐individual variability of performance in selected cognitive and physical tasks relying on speed processing and attention. Brain topology was measured via diffusion tensor imaging and quantified with clustering coefficients reflecting segregation and characteristic path lengths reflecting integration, respectively, based on graph theory methods.

**Result:**

After correcting for multi‐comparison, the high dual‐functional LDS group exhibited significantly higher integration between the inferior frontal and postcentral areas but lower integration between the frontal and temporary areas within the left hemisphere, compared to the other three low cognitive/physical stability groups.

**Conclusion:**

Integration in selective brain networks may contribute to maintaining the dual‐functional LDS in older adults.

